# Lower serum 25-hydroxyvitamin D3 concentration is associated with higher pain and disability in subjects with low back pain: a case–control study

**DOI:** 10.1186/s13104-019-4768-0

**Published:** 2019-11-08

**Authors:** Alireza Pishgahi, Neda Dolatkhah, Seyed Kazem Shakouri, Maryam Hashemian, Atefeh Amiri, Morteza Delkhosh Reihany, Fatemeh Jahanjou

**Affiliations:** 1 Physical Medicine and Rehabilitation Research Center, Aging Research Institute, Tabriz University of Medical Sciences, Emam Reza Hospital, Golgasht, Azadi Ave., Tabriz, Iran; 2 Metabolic Epidemiology Branch, Division of Cancer Epidemiology and Genetics, National Cancer Institute, Bethesda, USA; 3 Faculty of Medicine, Tabriz University of Medical Sciences, Tabriz, Iran

**Keywords:** Low back pain, 25-Hydroxivitamin D3, Functional disability, Pain intensity

## Abstract

**Objectives:**

Low back pain (LBP) is a common medical problem worldwide. The aim of this study is to evaluate the association between serum concentration of 25-hydroxivitamin D3 and functional disability in patients suffering from LBP in a sample of Azeri middle-aged subjects, North West of Iran.

**Results:**

In this case–control study, 63 eligible patients with LBP and 55 healthy subjects enrolled in the study. Peripheral venous blood was taken for evaluating the serum concentration of 25-hydroxivitamin D3. We recognized factors related with LBP by multiple regression analyses. The average serum 25-hydroxivitamin D3 concentration in case group was significantly lower than that of the matched controlled group (26.25 ± 15.95 vs. 34.20 ± 14.92, p-value < 0.01 respectively). Subjects with vitamin D deficiency or insufficiency were more likely to exhibit LBP than subjects with normal serum 25-hydroxivitamin D3 concentration [(OR = 2.388, 95% CI (1.114 to 5.119)]. According to the partial correlation analysis, there was a reverse correlation between serum 25-hydroxivitamin D3 concentration with functional disability measured by Modified Oswestry Questionnaire (r = − 0.307, p = 0.017) and also with pain intensity according to Visual Analogue Scale (VAS) score (r = − 0.268, p = 0.040) whilst adjusting for age, sex and body mass index (BMI).

## Introduction

Low back pain (LBP) is a common medical problem worldwide [1]. According to research, 65–80% of people have experienced LBP at least once in their life [2]. LBP is associated with difficulty in daily living activities, reduced social function, and diminished lifestyle quality [3].

Recently, Vitamin D deficiency and insufficiency have been pinpointed to be involved in many chronic disorders [4, 5] as well as chronic painful conditions [6, 7]. Meanwhile, the evidences concerning serum 25 hydroxyvitamin D concentration in subjects with and without LBP, and how vitamin D status affect pain severity in subjects with LBP seem to be inconsistent. There have been reports on a relationship between vitamin D deficiency and LBP incidence in literature. However, there has not been sufficient convicting evidence regarding the ideal serum concentration of 25 hydroxyvitamin D in this situation [8, 9].

Since the effect of vitamin D deficiency and vitamin D supplement vary among different populations [10–12] and considering the rising concern in vitamin D supplementation in LBP management, a broader understanding about the correlation between vitamin D status and severity of LBP is desirable. Accordingly, this study aims to evaluate serum concentration of 25 hydroxyvitamin D in patients suffering from LBP in a sample of Iranian population, northwest of Iran.

## Main text

### Methods

#### Patients

In this case–control study, 60 eligible patients with chronic LBP persistent for more than 3 months enrolled in the case group from the reference outpatient clinics of Tabriz University of Medical Sciences for further evaluation during a period of 3 months of Spring 2018. Inclusion criteria were as follows: age older than 20; BMI greater than 18.5 and LBP persistent for > 3 months. Patients were excluded if any of following conditions exists: lumbosacral deformities; intervertebral disc herniation; osteoporosis; rheumatoid arthritis; uncompensated renal and liver diseases. Pain-free control was selected from the same outpatient clinics if he/she matched a LBP subject already joined the study, according to the following criteria: age (± 5 years) and body mass index (BMI) (normal, overweight, obese). Exclusion criteria for controls were the same as for the case group.

A simple random sampling method was applied. Based on observations in a similar study [9], 6.2 and 6.9 were considered as the standard deviation (SD) of the case and control groups respectively and 3.6 for the effect size. Considering the type I error of 5% (α = 0.05) and type II error of 20% (β = 0.20; Power = 80%) and using a two-way test, the sample size was estimated as 53 individuals in each group. Considering 15% chance of loss, the sample size was estimated to be 60 in each group and 120 in total.

#### Physical activity

Physical activity of the participants was assessed by the short-form International Physical Activity Questionnaire (IPAQ) [13]. Three categories of physical activity were suggested: low, moderate, and high [14].

#### Vitamin D measurement

Fasting peripheral venous blood (3 ml) was collected from participants in both groups. The circulating concentration of 25-hydroxyvitamin D3 was measured by ELISA. All the steps were performed exactingly according to the instructions of the kits (Euroimmun, Germany) at the Emam Reza Hospital Clinical Biochemistry Lab (Tabriz, Iran). The results of the Euroimmun 25-OH Vitamin D ELISA correlate excellently with target values in the Vitamin D External Quality Assessment Scheme (DEQAS). The intra- and inter-assay variability coefficients were < 7% and < 9%, respectively.

#### Anthropometric measurements

The weight was measured by a Seca 813 digital scale at 8:00 a.m. in a fasting state. BMI was calculated via dividing the weight (kg) by the square of height (m^2^).

#### LBP assessment

Modified Oswestry Questionnaire was used for functional evaluation in individuals with LBP. This questionnaire included 10 questions regarding pain severity, LBP during self-care activities, lifting objects, walking, sitting, standing, sleeping, social life, and leisure time [15, 16]. LBP severity was also recorded by Visual Analogue Scale (VAS) in which zero referred to “No Pain” and 10 denoted “Very Severe Pain” [17].

#### Statistical measurement

Measurement data were reported as a mean  ±  SD. The two study groups were compared using Student’s t-test. Conditional logistic regression was used to approximation of odds ratio (OR) with 95% confidence intervals (CIs) for the risk of LBP. Each potential confounder that seems to be associated with outcome (physical activity) has been entered in the model. Finally, Partial correlation analyses were employed for analyzing correlation. The p < 0.05 was considered statistically significant. Statistical analysis was conducted using SPSS 17.0 software (SPSS Inc., Chicago, IL).

### Results

#### Patients

Totally 118 (73 female and 45 male) individuals were enrolled in this study, in which 63 had LBP (case group) and 55 were without LBP (control group). Five subjects were in the control were excluded in the analysis stage because of incomplete data and missed information. Details of demographic and anthropometric characteristics of participants are presented in Table 1. All the studied variables were normally distributed.

**Table 1:** Comparisons of demographic characteristics of participants in the case and control groups

Variables	Case group (N = 63)	Control group (N = 55)	p-value
Age, years	49.44 ± 12.77	50.02 ± 9.67	0.784
Sex			
Male	26 (41.26%)	29 (52.72%)	0.455
Female	37 (58.73%)	36 (65.45%)	
Education			
Illiterate/primary school	12 (19.04%)	16 (29.09%)	0.158
High school	22 (34.92%)	18 (32.72%)	
College	29 (46.03)	19 (34.54%)	
Job			
Workless	33 (52.38%)	24 (43.63%)	0.668
Employed	21 (33.33%)	27 (49.09%)	
Retired	9 (14.28%)	4 (7.27%)	
Weight (kg)	79.92 ± 12.30	75.27 ± 10.28	0.028
Height (cm)	165.50 ± 8.48	164.01 ± 9.08	0.336
BMI (kg/m^2^)	29.33 ± 5.05	28.10 ± 4.15	0.151
BMI category			
Normal	15 (23.80%)	25 (45.45%)	0.527
Over-weighted	29 (46.03%)	16 (29.09%)	
Obese	19 (30.15%)	14 (25.45%)	
Waist circumference (cm)	93.821 ± 8.72	94.985	0.522
Hip circumference (cm)	108.11 ± 15.99	108.67 ± 10.61	0.819
Waist to hip ratio	0.97 ± 0.93	0.87 ± 0.07	0.395
BMI			
Normal	15 (23.80%)	25 (45.45%)	0.527
Over-weighted	29 (46.03%)	16 (29.09%)	
Obese	19 (30.15%)	14 (25.45%)	
Physical activity			
Low	43 (68.3%)	25 (45.5%)	0.008
Moderate	20 (31.7%)	27 (49.1%)	
High	0 (00.00%)	3 (5.5%)	
Serum 25(OH) D (ng/ml)			
< 20	27 (42.85%)	8 (14.54%)	0.001
20 to 29.9	14 (22.22%)	15 (27.27%)	
≥ 30	22 (34.92%)	32 (58.18%)	

Values are mean ± SD OR number (%)

There were no significant differences between case and control groups in anthropometric measures except weight that was significantly higher in subjects with LBP than subjects in control group (p-value = 0.028). Normal waist circumference was observed in 3.6% of patients with LBP and 11% of matched healthy controls. Based on Pearson-correlation test results, there were significant inverse correlation between weight and serum concentration of 25 hydroxyvitamin D and also BMI and serum concentration of 25 hydroxyvitamin D in individuals with LBP (r = − 0.288, p-value = 0.022 and r = − 0.257, p-value = 0.042, respectively).

#### Vitamin D measurements

Serum concentration of 25 hydroxyvitamin D in case group was significantly lower than matched control group (26.25 ± 15.95 ng/ml and 34.20 ± 14.92 ng/ml, p-value < 0.01 respectively). In fact 42.85% and 22.22% of individuals with LBP and 14.54% and 27.27% of healthy controls had vitamin D deficiency and insufficiency, respectively.

The variables associated with LBP in simple analysis at significance level (p-value) of 0.25 (i.e. vitamin D deficiency or insufficiency and physical activity) were included in the multiple logistic regression method to explore the factors influencing LBP. The results of the adjusted analysis showed evidence of an increased risk of LBP in vitamin D deficiency or insufficiency (OR = 2.388, 95% CI 1.114 to 5.119). The values of crude OR and Adjusted OR are shown in Table 2.

**Table 2:** Multiple logistic regression analysis of LBP predictor

Variable	Case	Control	Crude OR (95% CI)	p-value	Adjusted OR (95% CI)^a^	p-value
Serum 25(OH) D (ng/ml)						
≥ 30	21	32	2.593 (1.231 to 5.463)	0.012	2.388 (1.114 to 5.119)	0.025
< 30	42	23				

^a^OR adjusted for physical activity

#### LBP characteristics: pain and disability

The mean score of the Modified Oswestry score for patients suffering from LBP was 31.12 ± 18.48 which is equal to “moderate disability”. Also the mean pain severity (VAS) score was 5.48 ± 2.12. There was a moderate, negative partial significant correlation between serum concentration of 25 hydroxyvitamin D and Modified Oswestry score (*r*(58) = − 0.307, *n* = 63, *p* = 0.017). However, zero-order correlations showed that there was a statistically significant, moderate, negative correlation between 25 hydroxyvitamin D serum concentration and functional disability of LBP (*r*(61) = − 0.336, *n* = 63, *p* = 0.007). There was also a negative partial significant correlation between serum concentration of 25 hydroxyvitamin D and VAS (*r* (57) = − 0.268, *n* = 63, *p* = 0.040) whilst controlling for age, sex and BMI. The zero-order correlations showed that there was a statistically significant, negative correlation between 25 hydroxyvitamin D serum concentration and VAS (*r* (60) = − 0.336, *n* = 63, *p* = 0.015), indicating that adjusting for age, sex and BMI did not alter the significance of the inverse associations between vitamin D status and either the levels of pain or of functional disability in patients with LBP.

### Discussion

There was a significant difference in serum concentration of 25 hydroxyvitamin D in patients with LBP in comparison to subjects without LBP. The serum concentration of 25 hydroxyvitamin D was significantly lower in patients with LBP and 42.85% and 22.22% of them had vitamin D deficiency and insufficiency, respectively. However, 14.54% and 27.27% of subjects without LBP showed vitamin D deficiency and insufficiency, respectively. According to the findings, subjects with lower serum 25 hydroxyvitamin D concentration (vitamin D deficiency or insufficiency) were 2.39 times more likely to exhibit LBP than subjects with normal vitamin D status. In a similar study in women with LBP, Hypovitaminosis D (25 hydroxyvitamin D < 40 ng/ml) was found in 49/60 patients with LBP (81%) and 12/20 (60%) of subjects without LBP, with an odds ratio of 2.97 [9]. However, Thorneby et al., in a cross-sectional study on individuals with chronic LBP, could not find any significant relationship between vitamin D status and LBP [18]. The reason for this variety of findings in studies regarding vitamin D status and LBP could be due to population variations across different societies. In the scientific literature, the Vitamin D status varies in various countries, even in various areas of the same country, in response to diversity in outdoor activity, exposure to the sunlight and dietary habits.

There were significant inverse correlations between weight as well as BMI and serum concentration of 25 hydroxyvitamin D in individuals with LBP. That means higher weight and BMI were associated with lower serum 25 hydroxyvitamin D concentration. Several studies [19–21], but not all [22], have suggested lower concentration of serum free 25 hydroxyvitamin D in obesity. Obesity has essential indications for the impression of vitamin D supplementation, and improvements in circulating 25 hydroxyvitamin D concentrations are commonly lower in obese/overweight subjects [23]. Effect of diet-induced obesity on biology of fatty tissue may contribute to the obesity-correlated decrease of free 25 hydroxyvitamin D. It has been shown that high-fat diet transcriptionally modifies Cyp2r1, which actively participates in vitamin D3 trapping and latter transformation to 25 hydroxyvitamin D [24]. These modifications are related to a reduction of free 25 hydroxyvitamin D in plasma [25].

In the current study, lower serum concentrations of 25 hydroxyvitamin D were associated with higher scores of disability questionnaire. The same pattern of relationship was also found for pain severity. Lower serum concentrations of 25 hydroxyvitamin D were associated with higher scores of LBP in VAS. In favor of findings in the current study, in a retrospective observational study in Turkey by Gokcek et al. [26] there was an inverse correlation between concentration of 25 hydroxyvitamin D and VAS score in patients with LBP (*r* = − 0.594, *p* < 0.001). In contrast, in a nested case–control study by Heuch et al. [8], no association was found between 25 hydroxyvitamin D status and risk of chronic LBP (OR per 10 nmol/l 25(OH)D = 1.01, 95% CI 0.97 to 1.06). What matters is that the interpretation of the findings of these studies will not be bias-free, regardless of the characteristics of the population studied. The prevalence and severity of vitamin D deficiency is very important in determining the association between the vitamin D status and LBP. In this regard, in the study of Brady et al. [27] on those with 25 hydroxyvitamin D < 30 nmol/l cholecalciferol supplementation led to a significant reduction in back pain disability. It seems that, these relationships are more conspicuous in those populations in which vitamin D deficiency is prevalent.

The exact mechanism linking vitamin D to pain is not completely clarified physiologically. It has been shown that vitamin D is associated with pain feelings through regulation of inflammatory cytokines [28] and modulation of sensory nerves [29]. Since there are some receptors of vitamin D present in the central nervous system, there have been theories proposed on relationship between vitamin D and fibromyalgia [30]. Vitamin D may also reduce the production of Prostaglandin E2 (PGE2) in fibroblasts which is a crucial factor for pain perception [31].

## Limitation

This study had some limitations, however. Firstly, finding representative control participants in the case–control design is not easy. Secondly, we did not determine the dietary intake of vitamin D in the participants. Nevertheless, a smaller quantity vitamin D is acquired from food than from sun exposure. Thirdly, the possibility of reverse confounding is another limitation of this study that should be considered. Severe back pain may limit ones physical activity; reduce one’s outdoor activity and sunshine exposure. Fourthly, the sample size was relatively small. Finally, causality cannot be confirmed because of retrospective design, and prospective and interventional studies are required to verify these findings.

## Data Availability

The datasets generated during the current study are available from the corresponding author on reasonable request.

## Abbreviations

BMI: body mass index
IPAQ: International Physical Activity Questionnaire
LBP: low back pain
OR: odds ratio
VAS: Visual Analogue Scale
